# Continued spring phenological advance under global warming hiatus over the Pan-Third Pole

**DOI:** 10.3389/fpls.2022.1071858

**Published:** 2022-11-24

**Authors:** Zhengjie Yan, Jinfeng Xu, Xiaoyi Wang, Zhiyong Yang, Dan Liu, Guoshuai Li, Huabing Huang

**Affiliations:** ^1^ College of Ecology, Lanzhou University, Lanzhou, China; ^2^ Center for the Pan-Third Pole Environment, Lanzhou University, Lanzhou, China; ^3^ State Key Laboratory of Tibetan Plateau Earth System, Environment and Resources (TPESER), Institute of Tibetan Plateau Research, Chinese Academy of Sciences, Beijing, China; ^4^ Heihe Remote Sensing Experimental Research Station, Key Laboratory of Remote Sensing of Gansu Province, Northwest Institute of Eco-Environment and Resources, Chinese Academy of Sciences, Lanzhou, China; ^5^ School of Geospatial Engineering and Science, Sun Yat-Sen University, Guangzhou, China; ^6^ Southern Marine Science and Engineering Guangdong Laboratory (Zhuhai), Sun Yat-Sen University, Zhuhai, China

**Keywords:** spring phenology, alpine vegetation, asymmetric warming, water availability, warming hiatus

## Abstract

The global surface temperature has witnessed a warming hiatus in the first decade of this century, but how this slowing down of warming will impact spring phenology over Pan-Third Pole remains unclear. Here, we combined multiple satellite-derived vegetation indices with eddy covariance datasets to evaluate the spatiotemporal changes in spring phenological changes over the Pan-Third Pole. We found that the spring phenology over Pan-Third Pole continues to advance at the rate of 4.8 days decade^-1^ during the warming hiatus period, which is contrasted to a non-significant change over the northern hemisphere. Such a significant and continued advance in spring phenology was mainly attributed to an increase in preseason minimum temperature and water availability. Moreover, there is an overall increasing importance of precipitation on changes in spring phenology during the last four decades. We further demonstrated that this increasingly negative correlation was also found across more than two-thirds of the dryland region, tentatively suggesting that spring phenological changes might shift from temperature to precipitation-controlled over the Pan-Third Pole in a warmer world.

## 1 Introduction

Vegetation phenology represents timing of the key vegetation developmental events in the seasonal cycle and is deemed as a sensitive indicator of climate change (Stocker et al., 2014). The ongoing climate warming has generally advanced spring phenology, delayed autumn phenology, and extended the growing season length (Menzel et al., 2006; Piao et al., 2006; Jeong et al., 2011; Gill et al., 2015). These changes in vegetation phenology are not only sensitive to climate change but also play a fundamental role in regulating regional carbon fluxes (Jin et al., 2016; Piao et al., 2017), water balance (Peñuelas et al., 2009), and energy exchange (Richardson et al., 2013). It is therefore imperative to assess the vegetation phenology changes and their underlying drivers to advance the understanding of how terrestrial ecosystems respond to changing climate (Badeck et al., 2004; Zhang et al., 2004; Pau et al., 2011; Tang et al., 2016; Piao et al., 2019; Shen et al., 2022).

The Pan-Third Pole encompasses Euro Asia high-land and its surroundings and covers the core region of the Belt and Road Initiative (Yao et al., 2017). The region distributes various ecologically fragile ecosystems known for high elevation and high aridity, and is therefore particularly vulnerable to drastic climate change. However, compared to the northern high latitude, assessment of vegetation phenology changes and its underlying mechanisms over the Pan-Third Pole has received much less attention. Previous studies focused on part of the Euro Asia high-land, have revealed general advances in spring phenology (Menzel et al., 2006; Shen et al., 2015; Shen et al., 2022), but the advancing magnitude differed among areas. For example, over the Tibetan Plateau, a series of research based on satellite data found an advancing trend in spring phenology from 1982 to the mid-1990s, but the advance slowed down after the mid-1990s (Yu et al., 2010; Chen et al., 2011; Piao et al., 2011; Shen, 2011; Shen et al., 2011). While a continued advance in spring phenology in the first decades of this century, was found in the western Tianshan Mountains, Ili Valley (Yang et al., 2022), and the northern Alps of European (Meng et al., 2021). What’s more, a significant slowing down of spring phenology during 2000-2011 were found in the lowland of the Pan-Third Pole (Fu et al., 2014), with even slight delaying trends detected in the south-western Tibetan Plateau (Shen et al., 2022) and high altitude (>2500 m) of Tianshan Mountains in central Asia (Ding et al., 2022), and French Alps (Asse et al., 2018).

To date, a comparative analysis of spatiotemporal change in spring phenology and its drivers over the Pan-Third Pole is still lacking. Previous studies show daytime maximum temperature (Tmax) and nighttime minimum temperature (Tmin) has a divergent impact on regional spring phenology (Piao et al., 2015; Shen et al., 2016). Precipitation is detected as the main driver in the dryland of Central Asia, with faster advancement in dryland spring phenology (Kariyeva and van Leeuwen, 2011; Kariyeva and van Leeuwen, 2012). In this way, the spatial divergence in temporal trend in spring phenology may be attributed to the spatial variation in Tmax, Tmin, or precipitation over Pan-Third Pole. Moreover, the global mean temperatures witnessed a widespread warming hiatus in the first decade of this century. A recent study, using several eddy covariance data and multi-remote sensed data, suggested that there is no widespread trend in spring phenology over the northern hemisphere during the warming hiatus (Wang et al., 2019). While all the eddy covariance data used in the analysis are distributed in North America or Europe, it remains unclear how the slowing down of warming impacts the spring phenology over the Pan-Third Pole.

Here, we examined spatiotemporal change in spring phenology and its underlying mechanisms over the Pan-Third Pole. First, we extracted the spring phenology in the Pan-Third Pole based on multiple satellite-based proxies and validated multiple satellite-based results by the eddy covariance data (EC). Then we examined change in spring phenology over the past four decades (1982–2015), with special attention to the warming hiatus period. We hypothesize that the asymmetric warming between daytime and nighttime temperature and change in precipitation might contribute to the spatial divergence in temporal trend in spring phenology over the Pan-Third Pole. This study aimed to (1) explore how spring phenology changed over the Pan-Third Pole during the warming hiatus, (2) determine the climatic factors regulating spring phenology, and (3) analyze whether those climate regulating factors changed as with climate change changes.

## 2 Material and methods

### 2.1 Study area

Pan-Third Pole covers the Euro-Asian highland, including Tibetan Plateau, Pamir, Hindu Kush, Tianshan, Iranian Plateau, Caucasus, Carpathians, and surrounding regions. The Pan-Third Pole covers more than 20 million km^2^ in area and is environmentally related to more than 2/3 of global humanity (Yao et al., 2017). Over the past 40 years, the Pan-Third Pole has experienced significant warming, far surpassing the global average, while this warming has witnessed a significant slowing down, and is known as warming hiatus (An et al., 2016; Huang et al., 2017).

The vegetation of the Pan-Third Pole mainly includes forests (south of the Tibetan Plateau, Alps 36.0% of the total region); grassland (Tibetan Plateau, Pamir, Tianshan Mountains, etc. 32.3% of the total region); cropland (Alps, Yunnan-Guizhou Plateau, 17.0% of the total region); shrub (Iranian Plateau, Kazak Hills 14.8% of the total region). To avoid the weak seasonal vegetation index signal of subtropical evergreen vegetation, the broad-leaved forest in southeast TP was not included in this study (0.1% of the total region).

### 2.2 Datasets

#### 2.2.1 Satellite-based vegetation proxies

To assess the spring phenology, we used two satellite‐derived vegetation proxies, including Normalized Difference Vegetation Index (NDVI) and Solar-induced Chlorophyll Fluorescence (SIF) from different sensors, to characterize spring phenology over the Pan-Third Pole. Specifically, we considered two NDVI datasets provided by Advanced Very High-Resolution Radiometer (AVHRR) sensors onboard NOAA satellites (Tucker et al., 2005) (hereafter NDVI3g) and the Moderate Resolution Imaging Spectroradiometer (MODIS) sensors onboard Terra and Aqua (Didan, 2015) (hereafter NDVIm). NDVI3g dataset was developed by the GIMMS group from the AVHRR sensors with a spatial resolution of one-twelfth of 1° (~10 km) and a 15‐day time step and is currently the longest continuous NDVI time series (1982–2015). NDVIm was extracted from MODIS MOD13A1 product (Collection 6), and has the 16-day time step, with a spatial resolution of ~500 m, and covers the period 2001–2015. SIF data which is a proxy for gross primary productivity, a daily SIF product (Köhler et al., 2015) was used to derive spring phenology. All the data sets are aggregated to a spatial resolution of 0.5° × 0.5° using bilinear interpolation to ensure consistency of analysis between the different products.

#### 2.2.2 Eddy covariance flux sites

We used sixteen eddy covariance observations to validate satellite-based spring phenology across the Pan-Third Pole. All the data were processed according to the standard protocol of the FLUXNET data set (Reichstein et al., 2005), e.g. data filtering, gap filling, and flux partitioning of those measurements. Noted eddy covariance observations are very sparse and mainly distributed in Europe and eastern TP.

#### 2.2.3 Climate data

To assess the impact of climate data on spring phenology, we compiled daytime maximum temperature, nighttime minimum temperature, and daily mean temperature, precipitation, and insolation with a spatial resolution of 0.5 × 0.5° covering the period 1982-2015 from Climatic Research Unit‐National Centers for Environmental Prediction (CRU‐NCEP) climate dataset (Viovy, 2018). Besides, to validate the robustness of temperature changes, we also compiled data from the Climate Research Unit, University of East Anglia (CRU) and National Weather Service National Centers for Environmental Prediction (NOAA NCEP) at the spatial resolution of 0.5 × 0.5° covering the period 1982-2015.

#### 2.2.4 Landcover data and dryland region

**Figure 1:**
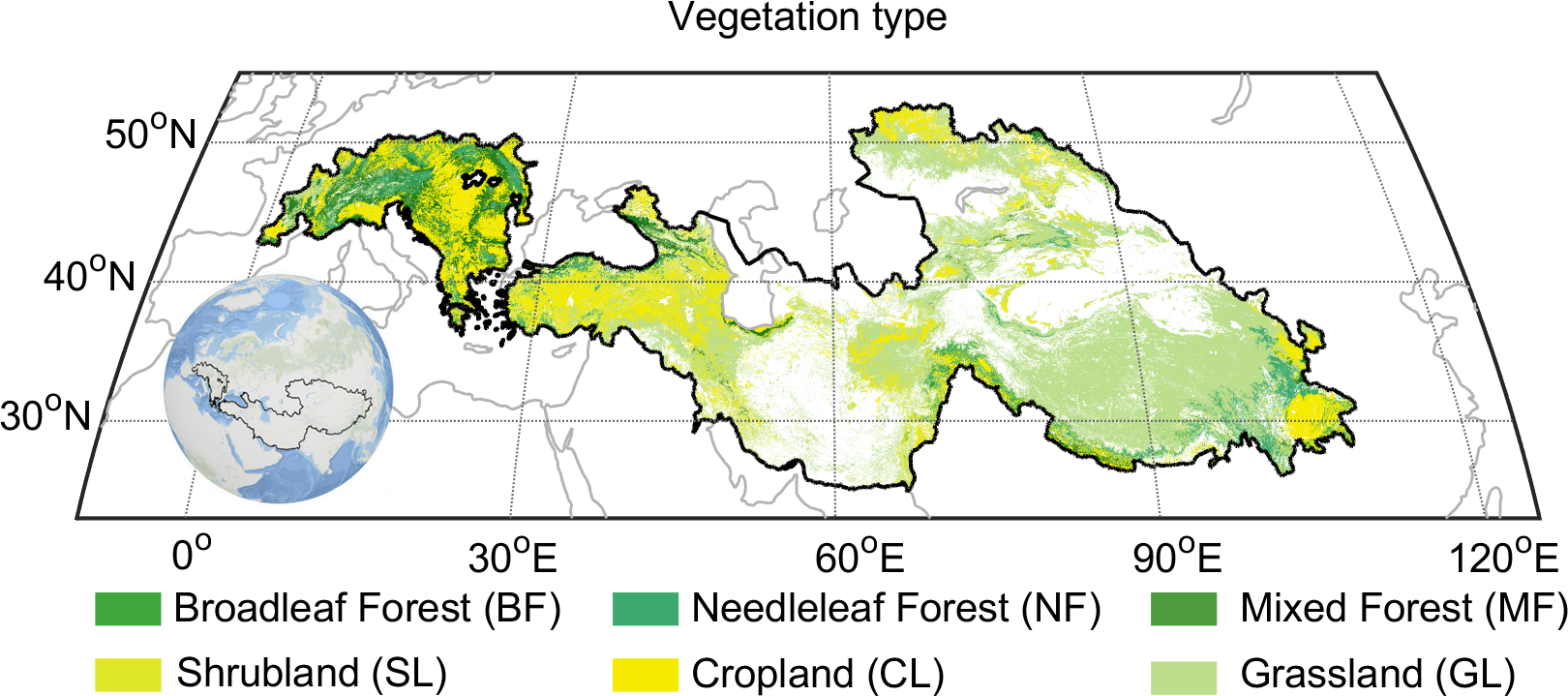
Distribution of vegetation type over the Pan-Third Pole. Vegetation type from the Global Land Cover 2000 (GLC2000) project uses the FAO Land Cover Classification System.

Vegetation‐type data for the Pan Third Pole were extracted from the GLC2000 landcover map generated by the European Commission’s Joint Research Centre and VEGETATION sensors on the SPOT4 satellite (Bartholome and Belward, 2005). The data provides multi-scale spatial resolution products. The original vegetation types were aggregated into the following six categories: broadleaf forest, mixed forest, needleleaf forest, shrubland, grassland, and cropland (**Figure 1**).

The aridity index, defined as the ratio of precipitation to evapotranspiration (AI), were calculated based on CRU datasets. The dryland region, with AI< 0.5, occupies 64.9% of the Pan-Third Pole (**Supplementary Figure 1**).

### 2.3 Analyses

#### 2.3.1 Determination of spring phenology based on multi-satellite vegetation proxies

Here we used consistent processing flow for different satellite datasets. Specifically, we first removed the snow effect before the processing (Wang et al., 2013). Here when the average temperature of five days falls below 0℃, the reflectance from satellite data might be affected by snow. We replaced those values with information from the nearest day without snow.

Then we fitted the original time series datasets (eg. NDVI, SIF) using the Gaussian function. The Gaussian function is usually expressed as (Cong et al., 2012):


$$VI(t)\;=\;a\;+\;b\;\times \;e\;-{\left(\frac{t-c}{2}\right)}^{2}$$

Where is the Julian date, *VI*(*t*) is the fitting *VI* value of date *t*, and *a*, *b*, *c*, and *d* are the fitting coefficients of the gaussian curve using the least square method. Gaussian curves are also known as “normal curves” with single peaks and symmetrical edges, so this fitting method is more suitable for the Pan Third Pole, where time series of vegetation proxies usually have only a single peak. Besides, to ensure the robustness of changes in spring phenology, we also used other four data filtering methods (cubic spline, HANTS, Timesat, and polyfit) to extract spring phenology, following (Cong et al., 2012; Cong et al., 2013; Cong et al., 2021).

Thirdly, we determined spring phenology as the date with the maximum change ratio as follows.


$$VI_{ratio}(t)\;=\;\frac{VI(t\;+1)\;-\;VI(t)}{VI(t)}$$

The type of *VI*(*t*) is the *VI* value at time *t, VI_ratio_* (*t*) is in the vegetation index of the relative change rate of time . In other words, the maximum value of *VI_ratio_* (*t*) is the time when the vegetation index time series has the maximum change. This maximum change ratio algorithm provides a dynamic threshold for phenology extraction and is widely adopted for large-scale phenology extraction (White et al., 2009).

#### 2.3.2 Statistical analysis

The spatiotemporal trends of spring phenology and temperature were calculated by a linear regression model using *t* test at the 95% significance level. To reveal the interannual associations between each climate driver and spring phenology, we calculated the partial correlation between spring phenology and one driver (e.g. Tmin), after controlling changes in other drivers (e.g. Tmax, precipitation and insolation). Here, the preseason climate drivers were considered, with the length of preseason for each pixel is calculated as the period before spring phenology with maximized partial-correlation coefficient between preseason climate driver (e.g. Tmin) and spring phenology (controlling the effects of other drivers e.g. Tmax, precipitation and insolation).

## 3 Results

### 3.1 Spatiotemporal changes in spring phenology over Pan Third Pole

We first depicted the spring phenology based on three vegetation proxies, including NDVI from GIMMS (NDVI3g), MODIS (NDVIm), and SIF from Global Ozone Monitoring Experiment-2 (GOME-2). To evaluate the robustness of our satellite-based spring phenology datasets, we resorted to sixteen eddy covariance sites recording daily Gross Primary Productivity (GPP) variation (**Supplementary Figure 2** and **Supplementary Table 1**), and extracted spring phenology as the date at which daily GPP rises above 15% of the multi-year daily GPP maximum (Richardson et al., 2010). Our results showed that satellite-derived spring phenology results are consistent with EC observations, with high correlations (*R*) ranging from 0.76 to 0.94, and the root-mean-square difference between 11 and 4 days (**Supplementary Figure 3**). Notably, the SIF-derived result has a better statistical performance in capturing phenology, with a smaller difference (3 ± 7 days) than that of the NDVI-derived result (16 ± 14 and 20 ± 19 days for NDVI3g and NDVIm). For example, for the CN-HaM sites, dominated by alpine grassland, and experiencing low annual temperature (2.4°C) and arid climate (annual precipitation ~ 370 mm), the NDVI-derived spring phenology (NDVI3g: 117 day of year, NDVIm: 135 day of year) diverge 28-10 days from that of SIF-derived results (145 day of year).

**Figure 2:**
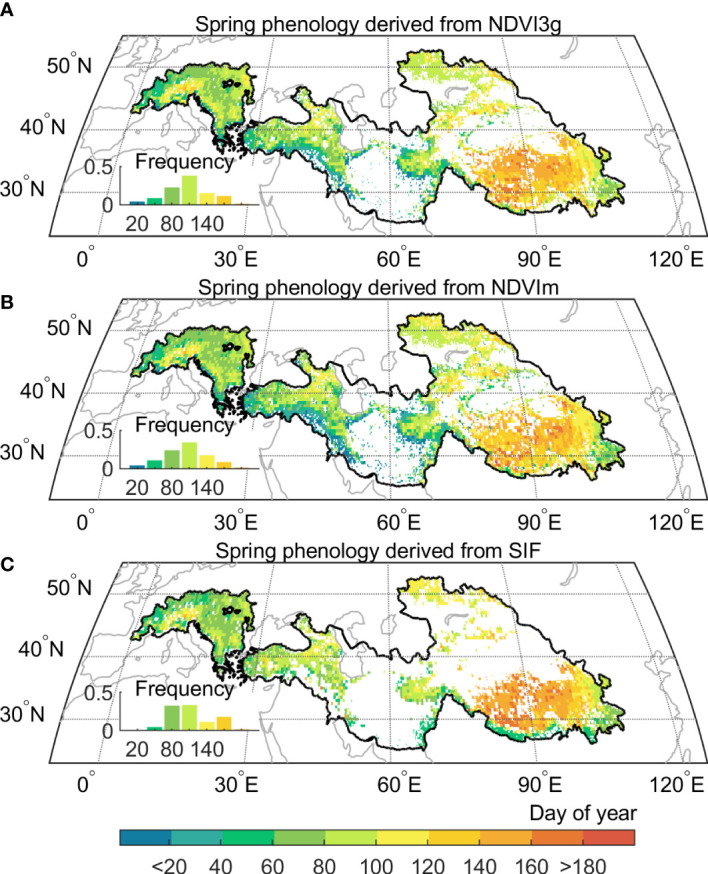
Multi satellites-based vegetation spring phenology in the Pan-Third Pole. **(A)** Spring phenology from GIMMS Normalized difference vegetation index (NDVI3g), and the period is from 1982 to 2015. **(B)** Spring phenology from Moderate Resolution Imaging Spectroradiometer (MODIS) C6 (NDVIm), and the time period 2001 to 2015. **(C)** Spring phenology from Solar‐Induced chlorophyll Fluorescence (SIF), and the period is from 2007 to 2015. All spatial distribution patterns represent the mean value of their period respectively.

The climatological spring phenology based on multiple proxies reveals a consistent spatial gradient from west to east, with earlier spring phenology in the Alps (109 ± 20 days) and Iranian Plateau (122 ± 32 days), and a much later one in the Tibetan Plateau (144 ± 24 days) and the Tianshan Mountains (140 ± 29 days) (**Figure 2**). Besides, the spring phenology delayed progressively along the increase of elevation, with the high elevation showing remarkable later spring phenology than the low one (138 ± 36 days > 3000 m vs. 104 ± 42 days< 3000 m). Here, since different proxies have a generally consistent pattern in spring phenological changes, we, therefore, use the longest records derived from NDVI3g hereafter, to reveal changes in spring phenology during 1982–2015, with a special emphasis on the changes after 1998, a period when the global warming hiatus occurred.

**Figure 3:**
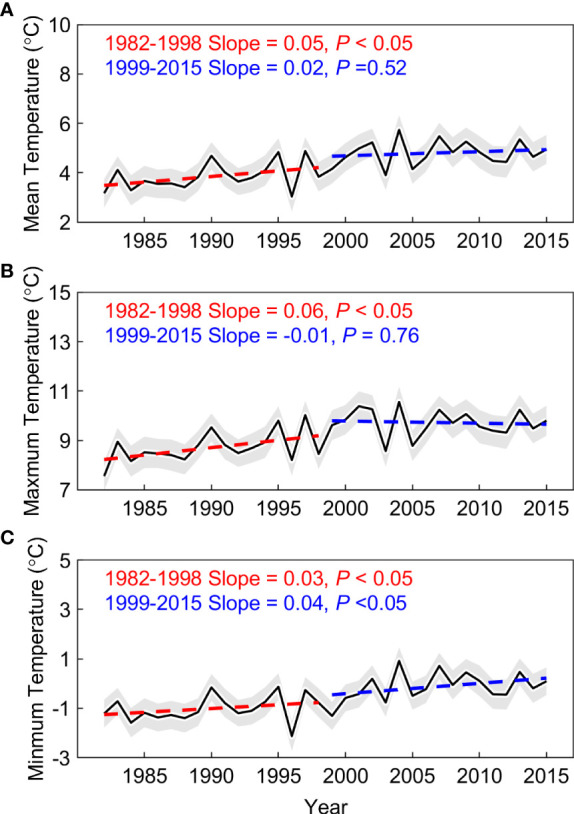
Multi satellites-based vegetation spring phenology in the Pan-Third Pole. **(A)** Spring phenology from GIMMS Normalized difference vegetation index (NDVI3g), and the period is from 1982 to 2015. **(B)** Spring phenology from Moderate Resolution Imaging Spectroradiometer (MODIS) C6 (NDVIm), and the time period 2001 to 2015. **(C)** Spring phenology from Solar‐Induced chlorophyll Fluorescence (SIF), and the period is from 2007 to 2015. All spatial distribution patterns represent the mean value of their period respectively.

The spring phenology significantly advanced at the rate of 4.5 days decades^-1^ (*P*< 0.01) across the Pan Third Pole during the whole period (1982–2015). Furthermore, we found that the advance rate of spring phenology is 5.9 days decades^-1^ (*P*< 0.01) before 1998, and this rate only slow down by 18.6% after 1998 and is still significant at the 99% significance level (4.8 days decades^-1^, *P*< 0.01). This significant advance rate was also observed during 2000–2015 using the NDVIm-derived spring phenology (4.6 days decades^-1^, *P*< 0.01), and was robust to the use of phenology extraction method (**Supplementary Figure 4**). Meanwhile, we resorted to multiple temperature records to examine the warming trend in the preseason period. Here the length of preseason for each pixel is searched as the period before spring phenology with maximized partial-correlation coefficient between preseason mean temperature and spring phenology (controlling the effects of precipitation and insolation). Evidence from three temperature datasets (CRUNCEP, CRU, and NOAA NCEP) shows a consistent slowing down of the warming rate (0.5 ± 0.1 vs. 0.02 ± 0.04°C decades^-1^ before and after 1998) over the Pan-Third Pole (**Figures 3** and **Supplementary Figures 5A1, A2**). A similar consistent slowing down was found for spring (March-May) temperature. We therefore documented a continued spring phenological advance during the warming hiatus period over the whole Pan-Third Pole.

**Figure 4:**
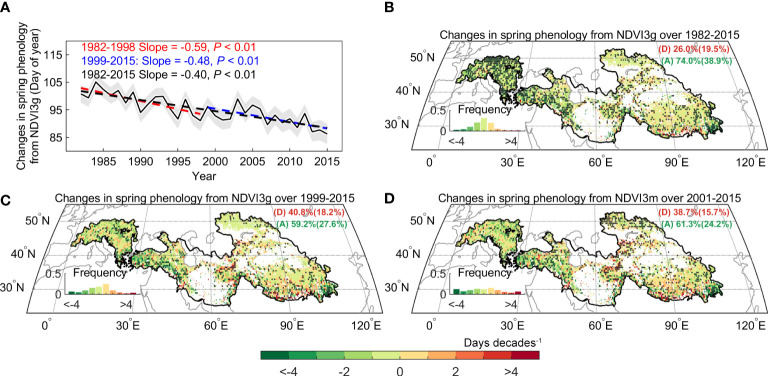
Spatial distribution of changes in spring phenology over the Pan-Third Pole. **(A)** Changes in regionally spring phenology across Pan-Third Pole over the period 1982–2015. **(B)** Changes in spring phenology derived from GIMMS Normalized difference vegetation index (NDVI3g) during 1982 – 2015 **(C)**, 1999 – 2015 **(D)**, and from Moderate Resolution Imaging Spectroradiometer (MODIS) C6 (NDVIm) during 2001 – 2015. The dots indicate the regions with a significant trend in spring phenology (*P<* 0.05).

Spatially, the continued advance of spring phenology is widely observed across the studied area (~74.0% and 59.2% for the period of 1982–2015, 1999–2015), which could be mainly found in the Alps, Iranian plateau, and east of Tibetan Plateau (**Figures 4**). Only 19.5% of the region had delayed spring phenology, which is scattered and distributed in part of the southern Alps, Pamir plateau, and the southern Tibetan Plateau. Most notably, using the aridity index defined as the ratio of precipitation to evapotranspiration (AI), the dryland region (AI< 0.5) which occupies 64.9% of the Pan-Third Pole has a larger advance rate (3.6 days decades^-1^, *P*< 0.01) than the sub-humid (AI > 0.5) region (3.4 days decades^-1^, *P*< 0.01). The results are also robust to the use of NDVIm-derived phenology (**Figure 4D**).

### 3.2 Drivers of temporal changes in spring phenology

**Figure 5:**
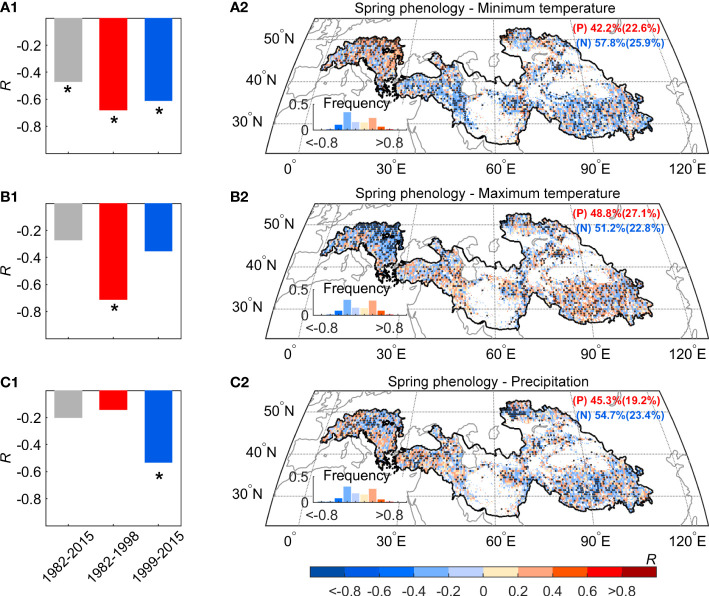
The relationship of the spring phenology with preseason Tmax, Tmin, and Precipitation during 1982–2015 over the Pan-Third Pole. Partial-correlation coefficients (*R*) between preseason Tmin **(A)**, Tmax **(B)** precipitation **(C)** after controlling for other factors. Here we also controlled insolation in the analysis. The dots indicate the regions with significant correlation coefficients (*P<* 0.05) and the asterisk indicate the regions with a significant correlation (P< 0.05).

**Figure 6:**
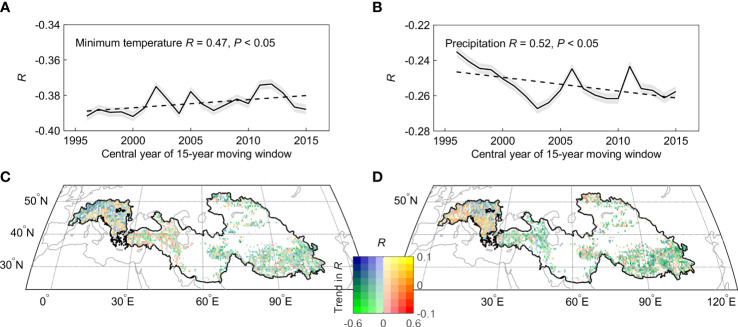
Spatial and temporal distribution of the partial correlation coefficients (*R*) between spring phenology and per-season climate. The 15-year sliding window were used to estimate trends in partial correlation between spring phenology and Tmin **(A, C)** and precipitation **(B, D)**.

We observed a widespread continuous advance in spring phenology, but this signal cannot be explained by changes in mean temperature. To reveal mechanisms behind the decoupling between mean temperature and spring phenology, we focused on two possible effects: the effect of asymmetric warming between daytime and nighttime, and the effect of precipitation, especially over the dryland region. According to temperature records based on three datasets (CRUNCEP, CRU, and NOAA NCEP), there exists widespread asymmetric warming between daytime and nighttime over the Pan-Third Pole. We found that only the daytime maximum temperature (Tmax) showed the warming hiatus, and there was a slight cooling trend after 1998 (-0.1°C decades^-1^, P > 0.1). While, the nighttime minimum temperature (Tmin) shows a continuous warming trend after 1998 (0.4°C decades^-1^, *P*< 0.05). We then detrended all the quantities and calculated the partial-correlation coefficients between spring phenology and preseason temperature (Tmax and Tmin). The inter-annual variation of spring phenology was significantly negatively correlated with preseason Tmin (*R* = -0.47, *P*< 0.05), but not with Tmax (*R* = -0.27, *P* > 0.1) (**Figures 5A1, B1**). Moreover, the significant effect of Tmin on spring phenology persists across the entire period (*R* = -0.68 and -0.61, *P*< 0.05, before and after 1998), with the increase of Tmin advancing spring phenology during the warming hiatus period using the metric of daily mean temperature. Our results are also robust to the analysis without the detrending of temperature variables. The continued significant effect of Tmin was also found using a 15-year sliding window (**Figures 6A, C**). In addition, spatial analysis between spring phenology and preseason Tmin reveals large spatial heterogeneity, with a significant negative correlation mainly distributed over the Tibetan Plateau, the Iranian Plateau, Pamirs, and Tianshan Mountain (**Figure 5A2**).

In addition, the Pan-Third Pole also includes part of the lowland with an elevation below 3000 m, such as the part of the region in the Alps and Iranian plateau. We also take a closer look at different altitude bands separately for regions above and below 3000 m and find a shift in the impact of Tmax on spring phenological changes for regions below 3000 m. Most notably, there is a shift in the Tmax impact in regions below 3000 m, and the negative impact shifted from significant (*R* = -0.72, *P*< 0.05) during the first period (1982-1998) to non-significant (*R* = -0.41, *P* > 0.1) during the second period (1998-2015) (**Figure 5B1**). By contrast, the negative effect of Tmax on spring phenological advance for high elevation is non-significant in both periods (*R* = -0.35 and -0.17, *P* > 0.1, before and after 1998) (**Figure 5B2**).

We then focused on the effect of moisture availability on spring phenological changes over the dryland region. We found that the partial correlation coefficient between spring phenology and preseason precipitation increased from non-significant (*R* = -0.14, *P* > 0.1) before 1998 to significant (*R* = -0.53, *P*< 0.05) after 1998 (**Figures 5C1**, **C2**). To test the robustness of such enhanced correlation, we further calculated the partial correlation between spring phenology and preseason precipitation using a 15-year sliding window (**Figures 6B, D**). Our results show that the absolute value of the negative impact of the preseason precipitation on spring phenology significantly increased across the entire period of 1982-2015. This increasingly negative correlation was also found across more than two-thirds of the dryland region. We therefore concluded that there is continuous control of Tmin and increasing importance of precipitation control on spring phenological changes.

## 4 Discussion

### 4.1 Continuous significant advance in spring phenology over the Pan-Third Pole

There is evidence from satellite and eddy-based results showing no advancing (or delaying) trends in spring phenology over the Northern Hemisphere during the warming hiatus (Wang et al., 2019). By contrast, our analysis shows that the spring phenology advanced significantly during the warming hiatus period over the Pan-Third Pole. This continuous significant advance in spring phenology can also be supported by regional studies. For example, based on the long-term *in situ* observations (site number = 21), Zheng et al. (2016) reveal the spring phenology of herbaceous plants significantly advanced during the first decades of this century. Shen et al. (2022) provided an overview of changes in spring phenology over the Tibetan Plateau and showed a continuous significant advance, with the advancing rate during the period 2000-2020 ranging from 2.1 days decades^-1^ (from CSIF) to 4.0 days decades^-1^ (from MODIS EVI and NDVI). Our analysis using NDVI3g (only have the data before the year 2015) also shows a significant advance at the rate of 3.8 days decades^-1^ over the Tibetan Plateau. Similar results were also found in Central Asia (Hyrcanian Forests of Iran). Kiapasha et al. (2017) showed that spring phenology advanced at the rate of 1.6 days decades^-1^, with more than 85% of the region showing non-significant trends.

To our best knowledge, this is the first study focused on the spring phenology over the Pan-Third Pole, and provided satellite-based evidence about how spring phenology changes and their controls, especially during the warming hiatus. Although we detect generally consistent significant advancing trends in spring phenology based on both NDVI3g and NDVIm datasets, there are still large uncertainties in the magnitude and/or sign of trends between different products (Peng et al., 2017; Moon et al., 2021; Ma et al., 2022). These inconsistencies could stem from the following factors: biological meaning (leaf emergence or plant photosynthesis), extraction methods (Cong et al., 2012), spatial and temporal resolution (different levels of mixed pixel effect, and different observation frequency) (Zhang et al., 2003; Zhang et al., 2009; Melaas et al., 2013; Shen et al., 2014; Tian et al., 2020; Tian et al., 2021), the BRDF effect (solar illumination angle and satellite view angle) (Morton et al., 2014; Ma et al., 2019; Petri and Galvao, 2019; Ma et al., 2020; Norris and Walker, 2020; Lu et al., 2022), and effects due to atmospheric (aerosols, clouds, and hazes) (Chen et al., 2004; Cai et al., 2017) or snow (Wang et al., 2013). For example, Yu et al. (2010) found a spring phenology started to slowing down in the mid-1990s. Zhang et al. (2013) documented continued advances, but with a substantially larger advance rate than *in situ* observations (Qi et al., 2006), possibly due to different processing procedure, e.g. ignoring the snow effect on non-growing season NDVI (Wang et al., 2013; Shen et al., 2013). The currently available longest eddy covariance data over Pan-Third Pole is only several years, and these measurements could then not be robustly used to validate satellite-derived trends. Therefore, the continued accumulation of field measurements is highly necessary to give a quantitative evaluation of the uncertainties in depicting spring phenology.

### 4.2 Continuous impact of minimum temperature on the spring phenology

In the cold area including the alpine and high-altitude regions, the temperature has been clearly deemed as the major driver for spring phenology (Park et al., 2016; Richardson et al., 2018). Higher temperatures during the preseason could accelerate physiological progress by increasing the heat accumulation rates (e.g. temperature exceeds a given threshold) for the leaf’s development (Körner and Basler, 2010; Luedeling et al., 2013). We showed that Tmin rather than Tmax facilitated changes in spring phenology. The strong control of Tmin on spring phenology was also found in recent analysis over the Tibetan Plateau (Shen et al., 2016), and the spring phenology is more sensitive to Tmin instead of Tmax in the central, eastern, and north-eastern parts of the Tibetan Plateau, with the increase in Tmin significantly advancing spring phenology by 4.2 days °C^-1^. The major climatic control of Tmin can also be found in tree-ring data over the eastern and south-eastern Tibetan Plateau, with the increase in Tmin advancing the initiates of xylem cell differentiation in trees (Yang et al., 2017). The increase in Tmin could effectively reduce the risk of deadly frosts (Inouye, 2008), and therefore allow some opportunistic species to leaf out earlier and thus be observed (Basler and Körner, 2012). Besides, the increase in Tmin could also influence available soil nutrients mediated by microbial activity (Basler and Körner, 2012; Heberling et al., 2019; Lee and Ibanez, 2021). Specifically, an increase in Tmin has the potential to remove the low-temperature restriction on microbial activity, and thus provide more available nitrogen (Heberling et al., 2019), which is beneficial to the dormancy release and growth recovery of plants. The significant impact of Tmin on spring phenological change was found in more than 76% of the high-elevational region (> 3000 m), suggesting a universe driver for the alpine region.

### 4.3 Increasingly importance of precipitation control on the dryland spring phenology

In contrast to temperature, the impact of precipitation on spring phenological changes has been understudied (Shen et al., 2011; Shen et al., 2015). Here we demonstrated that precipitation has a significant impact on spring phenology over the Pan-Third Pole during the warming hiatus period. This is because higher precipitation could be more likely to meet the water requirement for initiating leaf establishment, therefore advancing the spring phenology in the dryland region. Our analysis further provided satellite-based evidence that precipitation is playing an increasingly important role in spring phenology over the Pan-Third Pole dryland, with partial correlation changes from non-significant to significant. The significant impact of precipitation on spring phenological changes was also found in long-term manipulations, with the treatments of experimentally increased precipitation advancing spring phenology (leaf-out onset) in alpine meadows of the Tibetan Plateau (Ji et al., 2019). The strong influence of preseason (May–June) precipitation on the start of xylem cell differentiation was also found in tree ring analysis (Yang et al., 2017). Besides, previous studies also show spring phenology is sensitive to changes in precipitation in dry regions (Shen et al., 2015) and dry years (Ganjurjav et al., 2020).

Other factors should be considered in further analysis. For example, changes in snowmelt time (Wang et al., 2018), snowfall (Chen et al., 2015), soil nutrients (Thackeray et al., 2008; Xi et al., 2015; Yin et al., 2017; Fu et al., 2019) and grazing may impact spring phenology. Here, we did not consider winter chilling and photoperiod in the analysis, since the underlying mechanism over alpine grassland is unclear, but mounting evidence have shown winter chilling and photoperiod may influence tree phenology in lowland region (Cong et al., 2017).

## 5 Conclusions

In summary, we used multi-source remote sensing data to provide a systematic assessment of the spring phenology changes over the Pan-Third Pole. Our analysis reveals that asymmetric warming and increased water availability contributed to a significant advance in the spring phenology during the mean temperature warming hiatus period. Moreover, we also detected an increasingly important role of precipitation in spring phenology. Given that the Pan-Third Pole is projected to warm faster than the global average, the climate control of spring phenological changes might shift from temperature to precipitation over the Pan-Third Pole. Besides, the earlier start of the growing season may lead to enhanced vegetation growth (Gao et al., 2022), but can increase water scarcity during the summer (Lian et al., 2020). How the changes in spring phenology over Pan-Third Pole affect regional carbon and water flux still need further exploration.

## Data availability statement

The original contributions presented in the study are included in the article/**Supplementary Material**. Further inquiries can be directed to the corresponding authors.

## Author contributions

XW designed the research. XW and JF drafted the paper. JF and ZJY perform the data analysis. ZYY, DL, GL and HH contributed to the interpretation of the results and to the text. All authors contributed to the article and approved the submitted version.

## Funding

This study was supported by the Strategic Priority Research Program (A) of the Chinese Academy of Sciences (XDA19070303), the Science and Technology Major Project of Tibetan Autonomous Region of China (XZ202201ZD0005G01), the Second Tibetan Plateau Scientific Expedition and Research Program (2019QZKK0405, 2022QZKK0101), and the National Natural Science Foundation of China (41901136, 41801097).

## Acknowledgments

We sincerely thank the anonymous reviewers for their insightful comments. We would like to thank the Global Inventory Monitoring and Modeling System (GIMMS) group for the free use of NDVI data, the NASA EOSDIS Land Processes DAAC for the free use of NDVI data, and a free user-friendliness daily SIF dataset derived from Global Ozone Monitoring Experiment-2 (GOME-2) by Köhler et al., and Climatic Research Unit (University of East Anglia) and NCAS for the free use of CRUNCEP data, and National Weather Service National Centers for Environmental Prediction for the free use of NOAA NCEP data, and the Data Portal serving the FLUXNET community for the EC observation.

## Conflict of interest

The authors declare that the research was conducted in the absence of any commercial or financial relationships that could be construed as a potential conflict of interest.

## Publisher’s note

All claims expressed in this article are solely those of the authors and do not necessarily represent those of their affiliated organizations, or those of the publisher, the editors and the reviewers. Any product that may be evaluated in this article, or claim that may be made by its manufacturer, is not guaranteed or endorsed by the publisher.
