# Supplementary material for: Continued spring phenological advance under global warming hiatus over the Pan-Third Pole
Source: Front Plant Sci. 2022 Nov 24;13:1071858. doi: 10.3389/fpls.2022.1071858 (PMC9729745; doi:10.3389/fpls.2022.1071858)
Supplement: Supplementary file 1 [file DataSheet_1.docx]

Supplementary Material

# Supplementary Figure


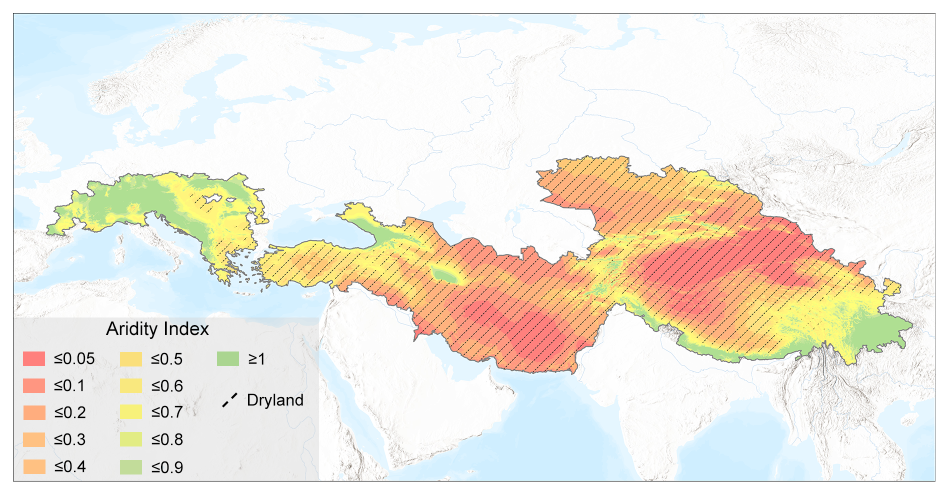


**Supplementary Figure 1. Spatial pattern of the aridity index (AI) in the Pan-Third Pole.** The Dryland region (AI < 0.5) was marked with a dashed line.


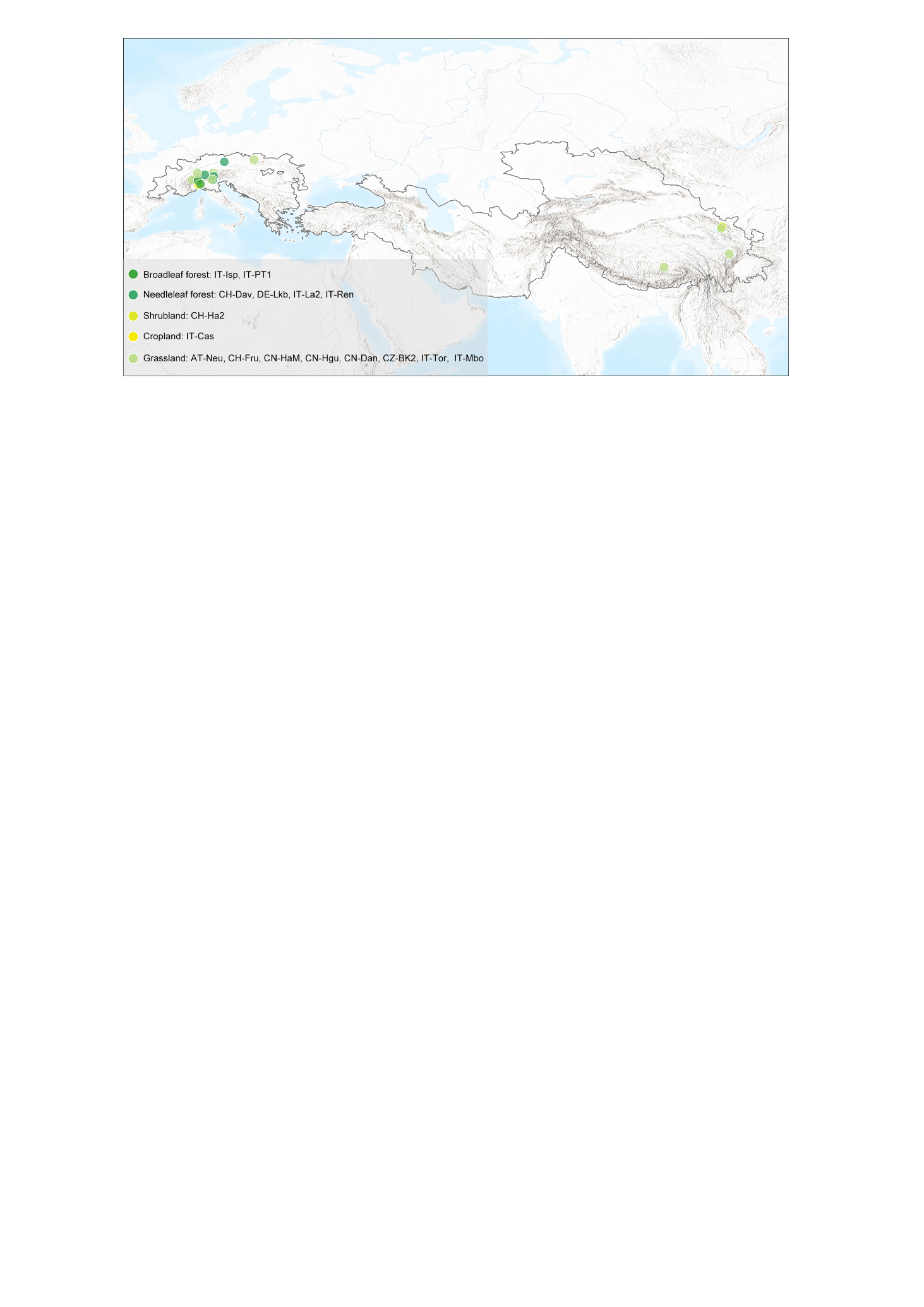


**Supplementary Figure 2. Distribution of the eddy covariance flux sites across the Pan-Third Pole (*N* = 16).**


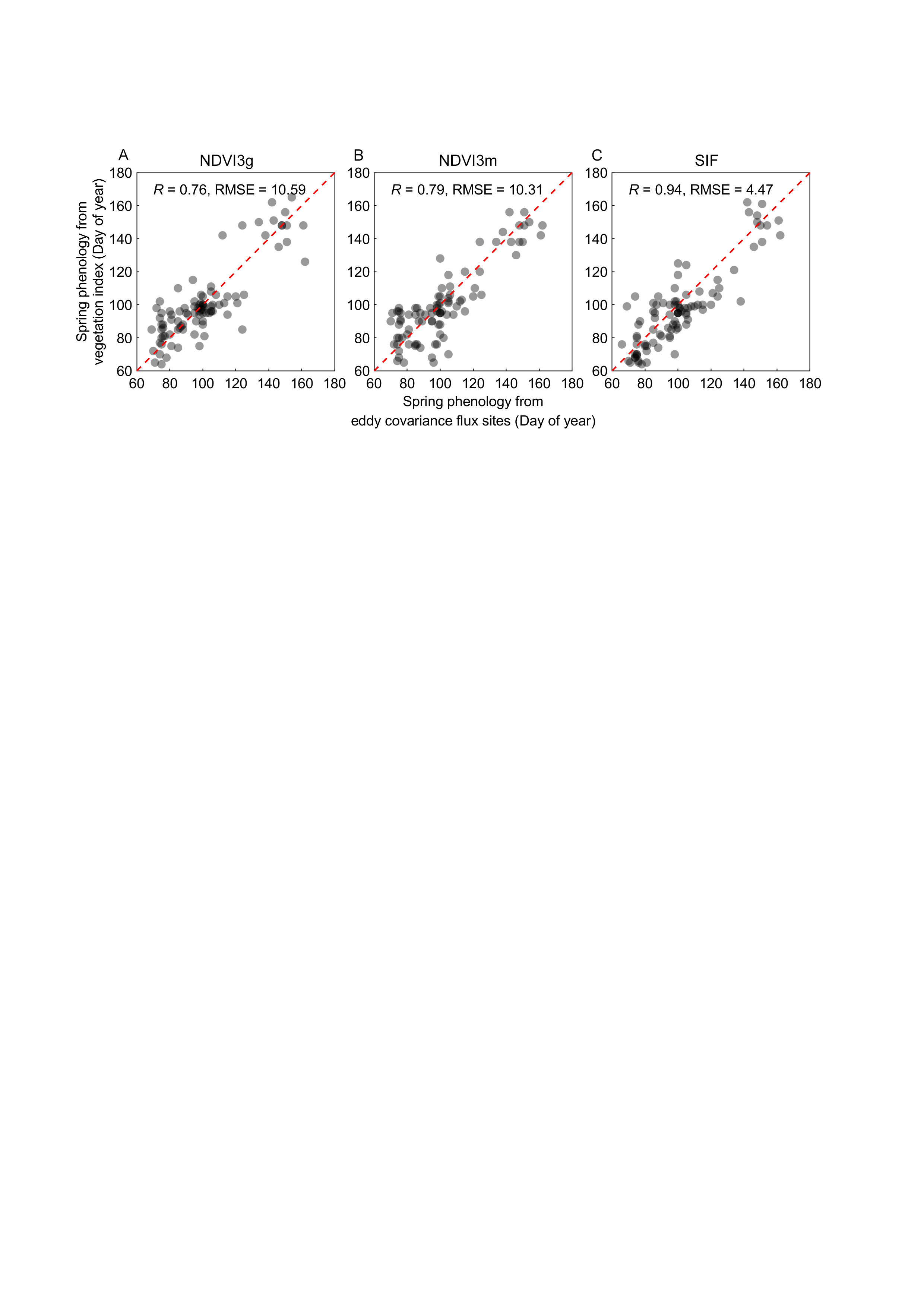


**Supplementary Figure 3.** **The comparison between eddy covariance-based spring phenology and satellite-derived results.** The 1:1 line were shown in dashed one. Performance of the satellite-based spring phenology was evaluated using both *R* and root mean square error (RMSE).


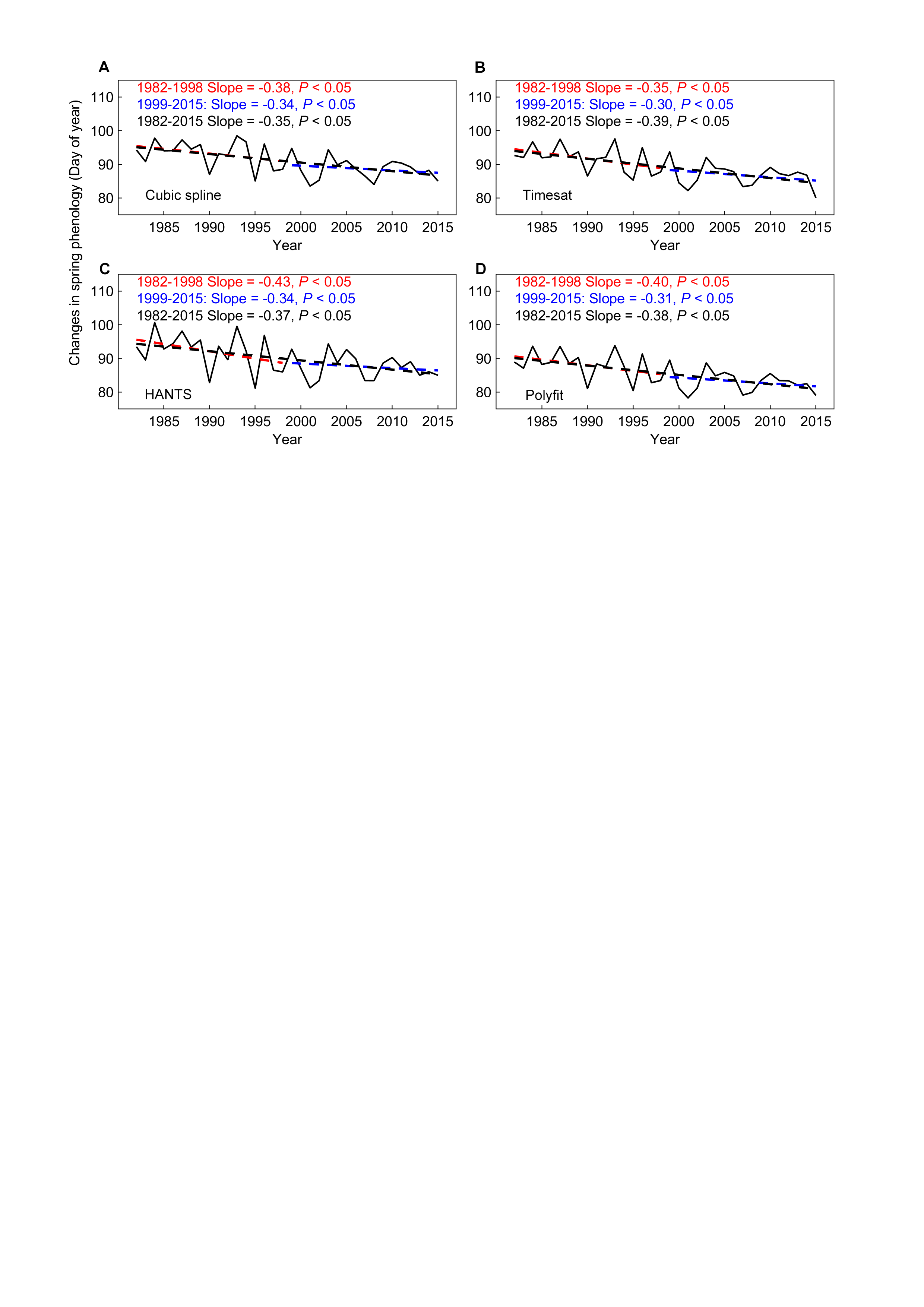


**Supplementary Figure 4. Changes in the spring phenology over the Pan-Third Pole based on NDVI3g using multiple data filtering methods.** The data filtering methods include Cubic spline **(A)**, Timesat **(B)**, HANTS **(C)**, and Polyfit **(D)**.

**
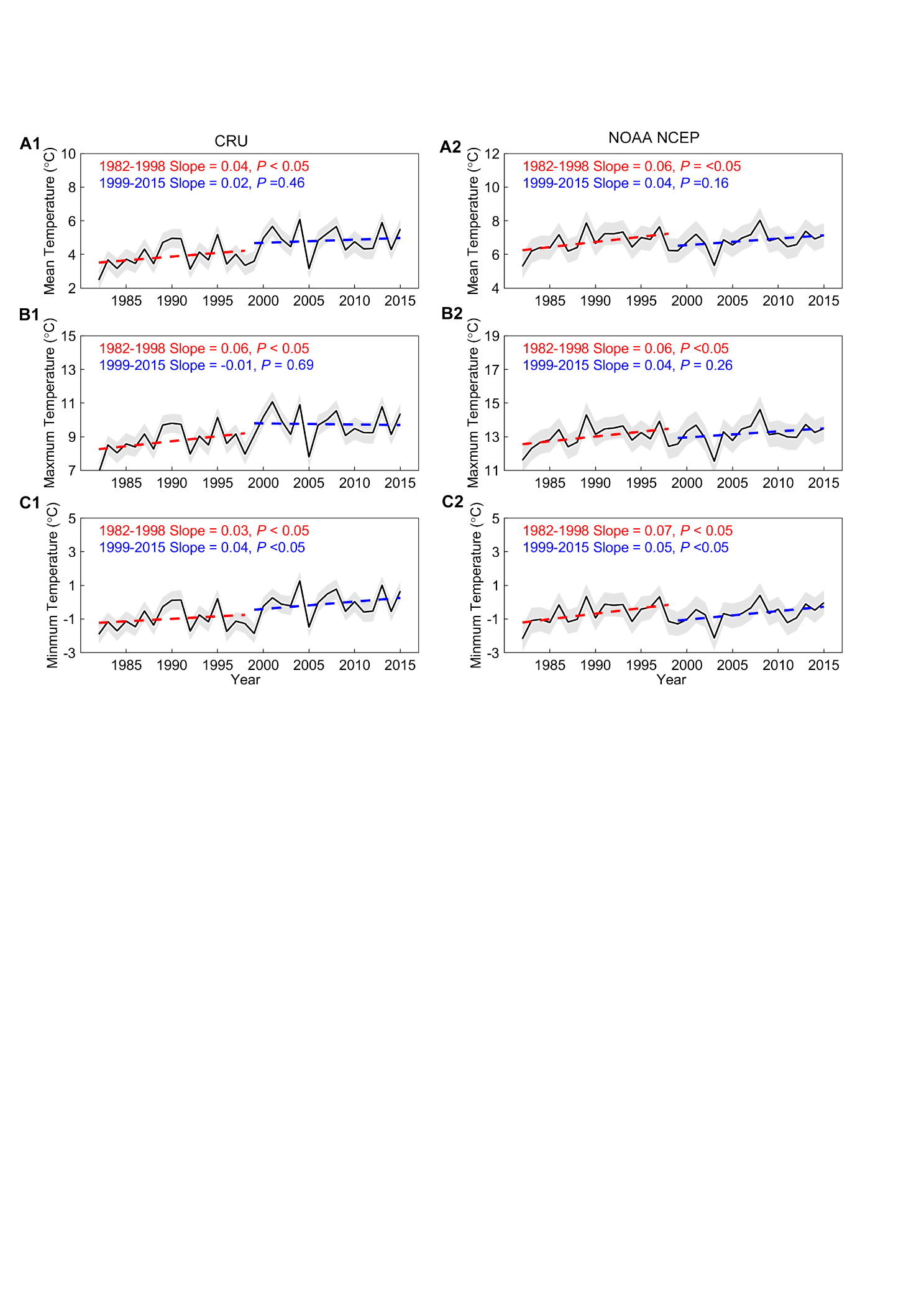
**

**Supplementary Figure 5. Changes in preseason temperature over the Pan-Third Pole during 1982–2015.** Preseason mean **(A)**, daytime maximum **(B)**, and nighttime minimum temperature **(C)** were evaluated based on two datasets (CRU, and NOAA NCEP).


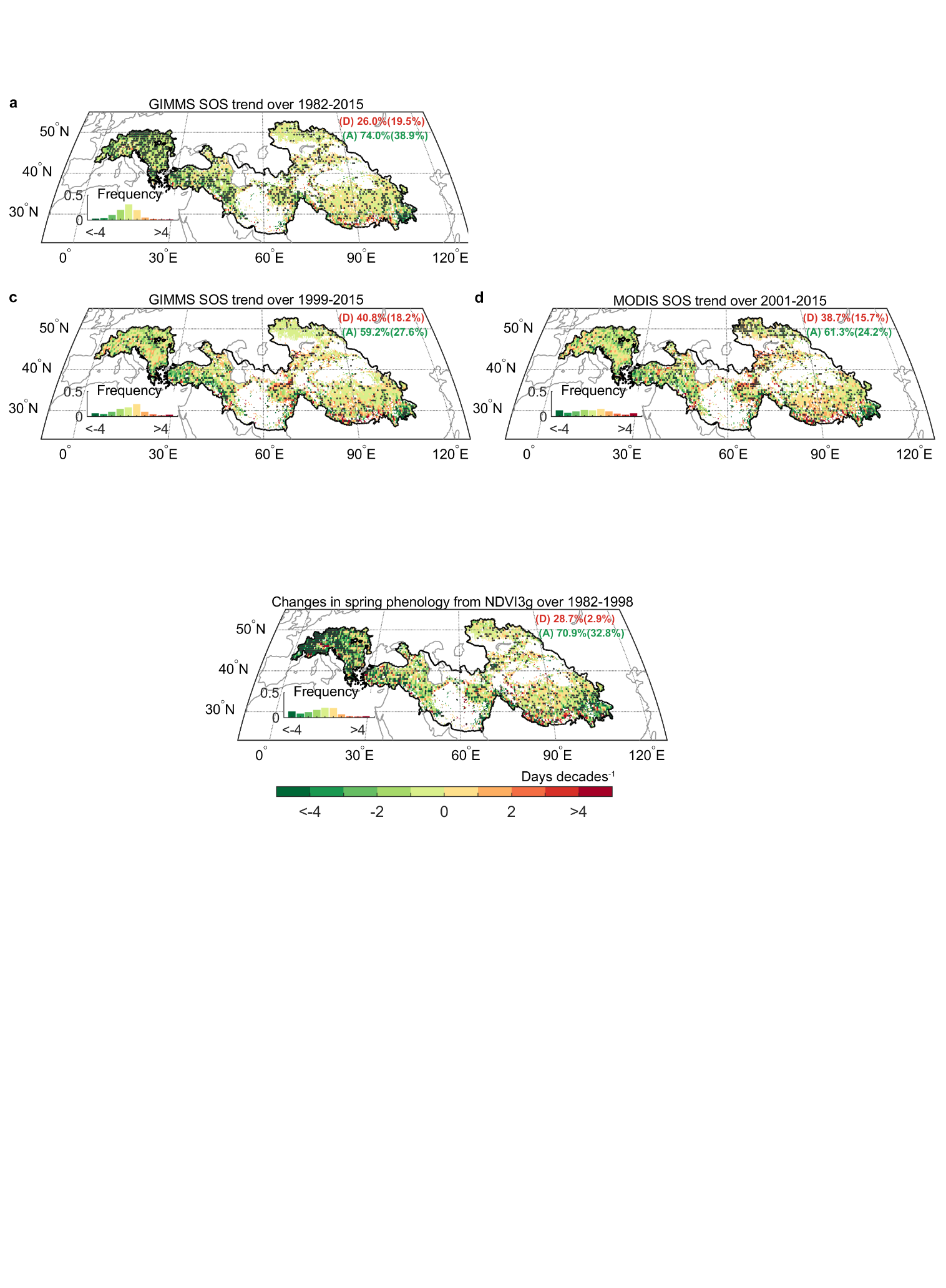


**Supplementary Figure 6. Spatial distribution of the changes in spring phenology derived from GIMMS Normalized Difference Vegetation Index (NDVI3g) in the Pan-Third Pole and the period is from 1982 to 1998.**

# Supplementary Table

**Supplementary Table 1. List of eddy covariance flux sites across the Pan-Third Pole used in our study.**

| Site | Vegetation type | Latitude(°N) | Longitude(°E) |
| --- | --- | --- | --- |
| AT-Neu | Grassland | 11.32 | 47.12 |
| CH-Fru | Grassland | 8.54 | 47.12 |
| CH-Dav | Broadleaf forest | 9.86 | 46.82 |
| CN-Dan | Grassland | 30.50 | 91.07 |
| CN-Ha2 | Shrubland | 37.61 | 101.33 |
| CN-HaM | Grassland | 37.37 | 101.18 |
| CN-Hgu | Grassland | 32.85 | 102.59 |
| CZ-BK2 | Grassland | 49.49 | 18.54 |
| DE-Lkb | Broadleaf forest | 49.10 | 13.30 |
| IT-Cas | Cropland | 45.07 | 8.72 |
| IT-Isp | Needleleaf forest | 45.81 | 8.63 |
| IT-La2 | Broadleaf forest | 45.95 | 11.29 |
| IT-Mbo | Grassland | 46.01 | 11.05 |
| IT-PT1 | Needleleaf forest | 45.20 | 9.06 |
| IT-Ren | Broadleaf forest | 46.59 | 11.43 |
| IT-Tor | Grassland | 45.84 | 7.58 |
